# *Hes1* Is Expressed in the Second Heart Field and Is Required for Outflow Tract Development

**DOI:** 10.1371/journal.pone.0006267

**Published:** 2009-07-17

**Authors:** Francesca Rochais, Mathieu Dandonneau, Karim Mesbah, Thérèse Jarry, Marie-Geneviève Mattei, Robert G. Kelly

**Affiliations:** 1 Developmental Biology Institute of Marseilles-Luminy, UMR 6216 CNRS-Université de la Méditerranée, Campus de Luminy, Marseille, France; 2 Inserm UMR 910, Faculté de Médecine, Hôpital d'Enfants de la Timone, Marseille, France; The University of Hong Kong, China

## Abstract

**Background:**

Rapid growth of the embryonic heart occurs by addition of progenitor cells of the second heart field to the poles of the elongating heart tube. Failure or perturbation of this process leads to congenital heart defects. In order to provide further insight into second heart field development we characterized the insertion site of a transgene expressed in the second heart field and outflow tract as the result of an integration site position effect.

**Results:**

Here we show that the integration site of the *A17-Myf5-nlacZ-T55* transgene lies upstream of *Hes1*, encoding a basic helix-loop-helix containing transcriptional repressor required for the maintenance of diverse progenitor cell populations during embryonic development. Transgene expression in a subset of *Hes1* expression sites, including the CNS, pharyngeal epithelia, pericardium, limb bud and lung endoderm suggests that *Hes1* is the endogenous target of regulatory elements trapped by the transgene. *Hes1* is expressed in pharyngeal endoderm and mesoderm including the second heart field. Analysis of *Hes1* mutant hearts at embryonic day 15.5 reveals outflow tract alignment defects including ventricular septal defects and overriding aorta. At earlier developmental stages, *Hes1* mutant embryos display defects in second heart field proliferation, a reduction in cardiac neural crest cells and failure to completely extend the outflow tract.

**Conclusions:**

*Hes1* is expressed in cardiac progenitor cells in the early embryo and is required for development of the arterial pole of the heart.

## Introduction

Cardiac progenitor cells of the second heart field (SHF) contribute to the rapid growth of the embryonic heart by adding cells to the poles of the heart tube [1]. At the arterial pole, SHF cells give rise to right ventricular and outflow tract (OFT) myocardium in addition to smooth muscle at the base of the great arteries [2]. The SHF originates in splanchnic mesoderm medial to the cardiac crescent from which the linear heart tube is derived and is characterized by the expression of *Fgf10*, *Isl1* and *Tbx1* [3]–[5]. The contribution of SHF derived cells to the heart is coordinated with that of cardiac neural crest cells, which are essential for dividing the embryonic OFT into the ascending aorta and pulmonary trunk [6]. Interactions between these two cell types in the pharyngeal region prior to their addition to the heart are critical for normal OFT development; perturbations in SHF or neural crest development results in failure to correctly elongate and divide the OFT, resulting in congenital heart defects including overriding aorta, double outlet right ventricle and common arterial trunk [6]–[8]. Despite recent advances in our understanding of OFT development, the molecular mechanisms maintaining SHF cells in a progenitor state and regulating their contribution to the elongating OFT remain poorly defined.

Notch intercellular signaling has been implicated in multiple aspects of heart morphogenesis including cushion development, trabecular growth, atrioventricular patterning, neural crest differentiation and arterial pole development [9]–[12] (reviewed in [13]). Mutations in the genes encoding the Notch ligand JAGGED1 or the NOTCH2 receptor have been identified in Alagille syndrome, associated with arterial pole defects including tetralogy of Fallot, and mutations in *Notch1* are associated with aortic valve disease [14]–[16]. The Hey (Hairy and Enhancer of Split related) family of basic-helix-loop-helix transcription factors is thought to mediate Notch signaling in the developing cardiovascular system [13]. *Hey2* mutant mice display arterial pole alignment and ventricular septal defects, associated with activation of the atrial program in ventricular myocardium [17]–[20]. Members of the related Hes (Hairy and Enhancer of Split) family of transcription factors are also activated by Notch signaling and play critical roles in development including maintaining progenitor cell populations, controlling binary cell fate decisions and regulating boundary formation [21]. *Hes1*, encoding a transcriptional repressor, plays central roles in cell proliferation and differentiation processes in multiple cell types and is required to maintain progenitor cells in an undifferentiated state [22]–[26].

Recently the *A17-Myf5-nlacZ-T55* (*T55*) transgene has been shown to be expressed in the SHF and OFT of the developing mouse heart as a result of an integration site position effect [27]. We have localized the *T55* transgene integration site upstream of *Hes1* and show that the transgene is expressed in a subset of *Hes1* expression sites, including pharyngeal mesoderm of the SHF. Although *Hes1* has not been previously implicated in heart development, mice lacking *Hes1* display arterial pole alignment anomalies, including overriding aorta and ventricular septal defects, common components of congenital heart disease in man. These defects are preceded by impaired deployment of cells of the SHF and cardiac neural crest revealing a requirement for Hes1 during OFT development.

## Results

### Characterization of the *T55* transgene integration site

**Figure 1:**
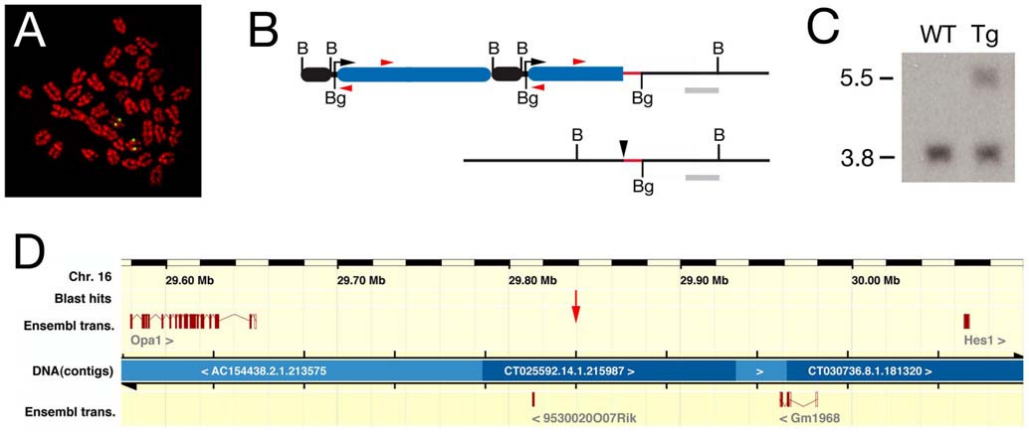
Molecular characterization of the *T55* transgene integration site. (A) Fluorescent in situ hybridization to metaphase chromosomes prepared from *T55* splenocytes showing transgene localization to chromosome 16 B2–B4. (B) Map of the *T55* integration site (top) and endogenous locus (bottom) showing the structure of the 3′ end of the transgene array (black box, *A17 Myf5* enhancer; blue box, *nlacZ* reporter gene) and the position of the inverse PCR primers (red arrowheads); B, *Bam*HI; Bg, *Bgl*II. Also shown are the flanking sequence isolated by inverse PCR (red) and the Southern blot probe (grey) used to identify the predicted 5.5 kb *BamHI* fragment in transgenic DNA in addition to a 3.8 kb wildtype fragment (C). (D) Ensembl map of the genomic region surrounding the integration site (red arrow) on chromosome 16, showing the position of the flanking genes *Opa1* and *Hes1* and intermediate gene predictions based on EST alignments.

The *Myf5 A17-nlacZ-T55* (*T55*) transgene is expressed in the SHF and OFT as a result of an integration site position effect [27]. In order to identify new genes expressed in the SHF, we characterized the transgene integration site. Using fluorescent in situ hybridization (FISH) the insertion site was mapped to chromosome 16 B2–B4 (Fig. 1A). An inverse PCR approach with primers in the 5′ region of the *nlacZ* reporter gene was used to isolate a transgene-integration site junction fragment containing 256 bp of flanking sequence (Fig. 1B). Southern blot and PCR analysis confirmed the presence of this junction specifically in DNA from mice carrying the *T55* transgene (Fig. 1C, data not shown).

**Figure 2:**
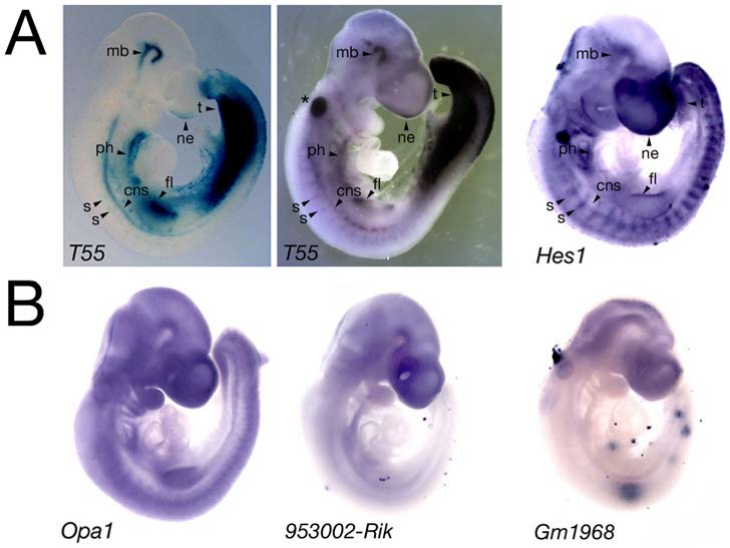
Comparison of transgene and integration site gene expression profiles. (A) E9.5 embryos after X-gal staining (*T55* embryo, left) or in situ hybridization with a lacZ (*T55* embryo, middle) or *Hes1* riboprobe (right). Overlapping expression sites include the ventral pharyngeal region (ph), forelimb (fl), ventral neural tube (cns), segmental intersomitic region (s), midbrain (mb), tail region (t) and nasal ectoderm (ne). Asterisk indicates *lacZ* riboprobe trapping in the otic vesicle. (B) Expression profiles of *Opa1* and two predicted genes mapping to the intergenic region at E9.5.

**Figure 3:**
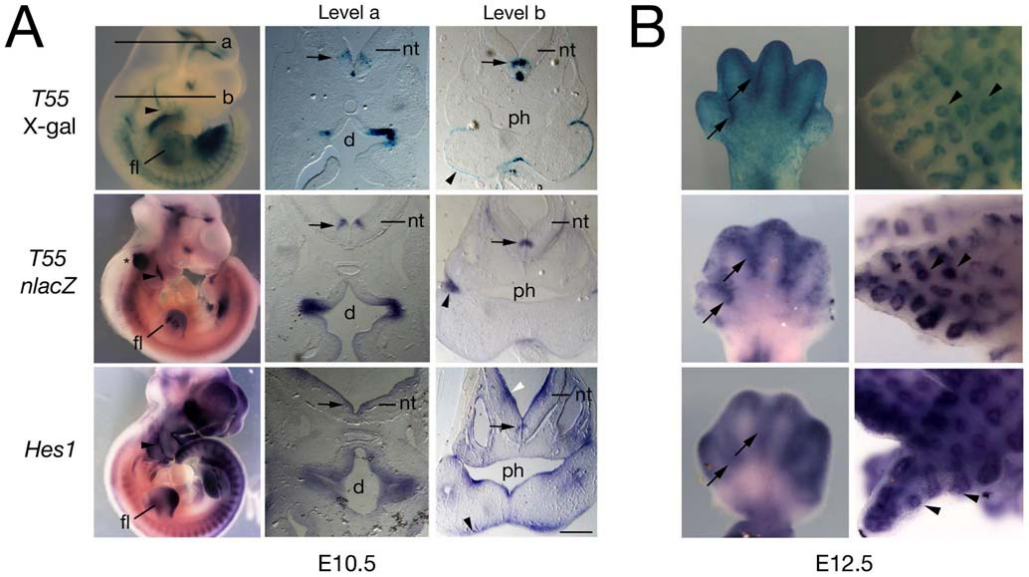
Comparison of *T55* and *Hes1* expression during embryonic development. (A) E10.5 wholemount embryos (left) and cryosections after X-gal staining (top) or in situ hybridization with a *lacZ* (T55 embryo, middle) or *Hes1* riboprobe (bottom). The transgene is expressed at a subset of sites of *Hes1* expression including the forelimb (fl), pharyngeal region (including ectoderm, arrowheads), neural tube (nt), including a specific population of neurons in the ventral neural tube (arrows) and diencephalon (d); ph, pharynx. Asterisk indicates probe trapping in the otic vesicle. (B) E12.5 forelimb (left) and lung (right) after X-gal staining (top) and *nlacZ* (middle) or *Hes1* (bottom) in situ hybridization, showing similar transgene and *Hes1* expression in the interdigital region (arrows) and pulmonary epithelium (arrowheads). Scale bar: 200 µm.

A nucleotide BLAST search using the 256 bp flanking sequence revealed that this sequence mapped to chromosome 16 B2, consistent with the FISH localization (Fig. 1D). The insertion site junction lies between two genes, Optic Atrophy 1 (*Opa1*), encoding a mitochondrial dynamin-related GTPase, and Hairy/Enhancer of Split 1 (*Hes1*), encoding a basic-helix-loop-helix containing transcriptional repressor, situated 258 kb and 224 kb from the isolated sequence respectively. *Opa1* and *Hes1* are part of a 4 gene synteny block centered on *Hes1* that is conserved in zebrafish. The expression profiles of *Hes1* and *Opa1*, together with those of 4 EST sequences and 2 predicted genes mapping between *Hes1* and *Opa1*, were evaluated by wholemount in situ hybridization at E9.5 (Fig. 2). *Hes1* transcripts showed a regionalized expression profile overlapping in distribution with the *T55* transgene (Fig. 2A, Fig. 3), including pharyngeal, forelimb, tail, intersomitic, neural tube, midbrain and nasal ectoderm expression sites (Fig. 2A). In contrast, *Opa1* transcripts were broadly expressed in the embryo and riboprobes detecting EST sequences between *Opa1* and *Hes1*, including the predicted genes 9530020007RIK and GM1968, revealed either low-level expression in the anterior region of the embryo or no expression (Fig. 2B). Together, these results suggest that *Hes1* may be the endogenous target of the cis-regulatory sequences trapped by the *T55* transgene.

**Figure 4:**
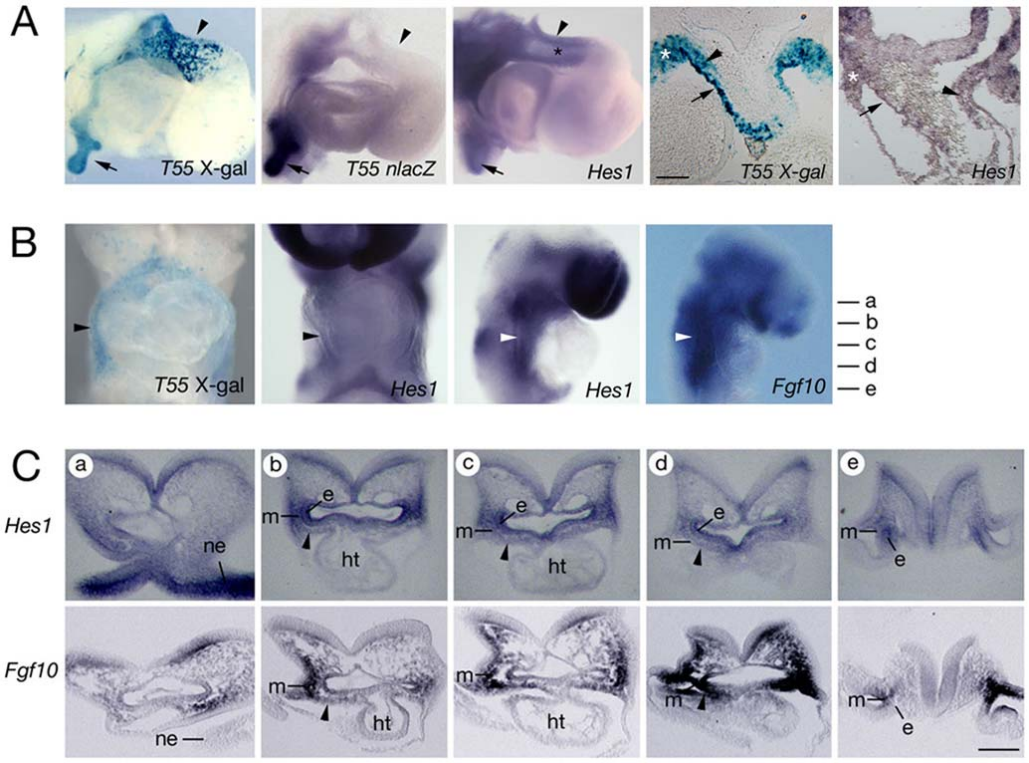
*Hes1* is expressed in the second heart field. (A) Comparison of transgene and *Hes1* expression in right lateral views of hearts after X-gal staining and in situ hybridization at E10.5 (left three panels). X-gal positive cells are observed in the distal OFT wall (arrowhead) and pulmonary endoderm (arrow); *nlacZ* transcripts are observed in pulmonary endoderm but not the OFT. Low-level Hes1 transcript accumulation is observed in the distal OFT (black asterisk). Transverse sections (right two panels) show β-galactosidase and low-level *Hes1* transcript accumulation in the pericardial region (white asterisks), superior OFT wall (arrow) and mesenchymal cells in the OFT (arrowhead). (B) At E8.5 X-gal and *Hes1* positive cells are observed in ventral and right views in the pericardial wall (black arrowhead) and pharyngeal mesoderm (white arrowheads) where *Fgf10* transcripts also accumulate. (C) E8.5 sections at the levels indicated in (B), showing *Hes1* transcripts in neuroepithelium (ne), endoderm (e) and mesoderm (m) lateral and ventral to the pharynx, including the SHF (arrowheads), compared with *Fgf10* expression. Note that caudally (level e) *Hes1* and *Fgf10* transcripts are observed in endoderm and mesoderm respectively. ht, heart tube. Scale bars: (A, C) 100 µm.

Additional gene expression studies were carried out to test this hypothesis. The distribution of *Hes1* transcripts was compared with that of *nlacZ*, together with β-galactosidase expression, in *T55* embryos at E10.5 and E12.5. At E10.5, *T55* and *Hes1* expression overlapped in pharyngeal epithelia, the pericardial region and the forelimb (Figs 3A, 4A). *T55* transcripts accumulated in a subset of *Hes1* expression sites in the central nervous system, including specific populations of neurons in the brain and ventral neural tube (Fig. 3A). At E12.5, expression of the transgene and *Hes1* was observed in the interdigital region of the developing limb-buds and pulmonary endoderm (Fig. 3B). These experiments revealed that the *T55* transgene and *Hes1* are co-expressed in a subset of *Hes1* expression sites.

### *Hes1* is expressed in the second heart field

In *T55* embryos, β-galactosidase activity is detected in the superior wall of the OFT and contiguous pericardial mesoderm at E10.5 [27]; *nlacZ* transcripts in *T55* positive embryos accumulate in pericardial mesoderm but were not observed in the myocardial OFT wall. β-galactosidase activity in the OFT wall is thus the result of either extremely low-level transcription or β-galactosidase perdurance from prior transcription in OFT progenitor cells (Fig. 4A). At this timepoint *Hes1* transcripts accumulate in pericardial mesoderm; after extensive staining, *Hes1* transcript accumulation was detected in cells in the superior wall of the OFT (Fig. 4A). *Hes1* transcripts also accumulate at low-level in cells likely to correspond to cardiac neural crest cells in the distal OFT; *T55* transgene expression is also observed in these cells (Fig. 4A). Analysis of earlier developmental timepoints revealed that the *T55* transgene is expressed in splanchnic mesoderm dorsal to the heart tube at E8.5 (Fig. 4B) [27]. Endogenous *Hes1* transcripts were observed in the dorsal pericardial wall and in mesoderm lateral to the pharynx, in addition to pharyngeal epithelia and neuroectoderm. The expression of *Hes1* in pharyngeal mesoderm overlaps with that of *Fgf10*; however, the mesodermal expression domain of *Fgf10* extends further, both in a posterior direction and into cranial mesoderm, than that of *Hes1* (Fig. 4C). *Hes1* is therefore coexpressed with *Fgf10* in pharyngeal mesoderm dorsal to the early heart tube, including the region of the SHF.

### Heart defects in *Hes1* mutant mice

**Figure 5:**
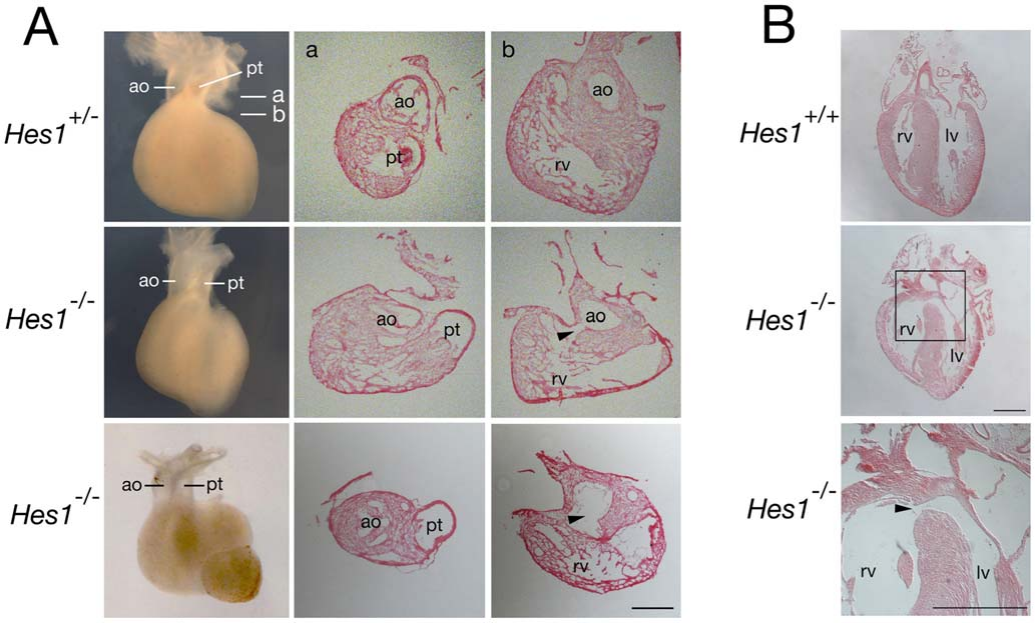
Congenital heart defects in *Hes1* mutant mice. (A) E15.5 hearts in ventral wholemount views (left) and cryostat sections at the levels indicated showing a normal configuration of the ascending aorta (ao) and pulmonary trunk (pt) in *Hes1* ^+/−^ hearts (top) and a dextraposed aorta overriding a ventricular septal defect (arrowhead) in *Hes1* ^−/−^ hearts (middle (mild) and bottom (severe)). Note that the aorta in the bottom heart symmetrically overrides the ventricular septal defect. (B) Paraffin sections of E18.5 hearts in a frontal plane showing a ventricular septal defect (arrowhead) in a *Hes1* ^−/−^ (middle and bottom) but not *Hes1* ^+/+^ heart (top). lv, left ventricle; rv, right ventricle. Scale bar: (A) 200 µm; (B) 500 µm.

*Hes1* mutant mice die during late fetal development with defects affecting neural, eye, pancreas, pituitary and lung development, among other organs [22]–[26]. This phenotype reflects the critical role of Hes1 in maintaining progenitor cell populations and regulating differentiation during organogenesis [22]. In order to investigate whether Hes1 is required for SHF development, we analyzed cardiac development in *Hes1* mutant mice. SHF defects affect OFT formation and arterial pole alignment [1], [4], [7] and embryos were scored at E15.5 for abnormalities in ventriculoarterial alignment. During normal development, the ascending aorta obtains an independent connection with the left ventricle and the pulmonary trunk with the right ventricle. 21/127 *Hes1* ^−/−^ embryos were recovered (expected 32/127; p>0.05)) and 8/21 (38%) mutant embryos displayed an externally visible dextraposed ascending aorta; this phenotype was not observed in *Hes1* ^+/−^ or *Hes1* ^+/+^ embryos (Fig. 5A). Histological sections of selected embryos revealed that the aorta failed to obtain a dorsal position with respect to the pulmonary trunk resulting in a ventricular septal defect and overriding aorta (observed in 3 out of 4 *Hes1* ^−/−^ hearts with a dextraposed aorta that were sectioned). The extent to which the aorta overrides the right ventricle was <50% in the hearts scored (Fig. 5). Overriding aorta and ventricular septal defects were not observed in 1 of the 4 sectioned hearts with a dextraposed aorta or in 6 additional *Hes1* ^−/−^ hearts without an externally visible dextraposed aorta. No defects were observed on histological analysis of 5 *Hes1* ^+/−^ and 5 *Hes1* ^+/+^ hearts. Loss of Hes1 therefore results in defects in normal alignment of the aorta and left ventricle during cardiac remodeling in a significant fraction of mutant embryos.

**Figure 6:**
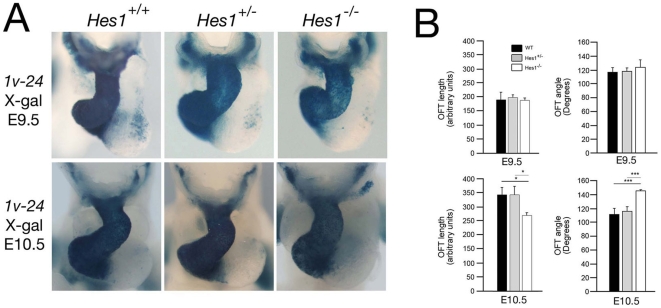
Outflow tract morphogenesis in *Hes1* ^−/−^ embryos. (A) Ventral whole mount views of X-Gal stained hearts from E9.5 and E10.5 *Hes1* ^+/+^, *Hes1* ^+/−^ and *Hes1* ^−/−^ embryos carrying the *Mlc1v-nlacZ-24* transgene. (B) Histograms showing OFT length measurements and the angle between the proximal and distal region of the OFT from *Hes1* ^+/+^, *Hes1* ^+/−^ and *Hes1* ^−/−^ hearts at E9.5 and E10.5. At E10.5, but not E9.5, *Hes1* ^−/−^ embryos display a shorter, straighter OFT (*, p<0.05; ***, p<0.001, Student's t-test).

The etiology of this defect was analyzed by examining *Hes1* ^−/−^ embryos at earlier stages of heart development. In order to visualize SHF progenitor cells and OFT development in mutant embryos, we crossed *Hes1* ^+/−^ mice with the *Mlc1v-nlacZ-24* transgenic line, in which transgene integration upstream of the *Fgf10* locus results in expression in pharyngeal mesoderm, including cells of the SHF, OFT and right ventricle (Fig. 6A) [3]. We analyzed OFT morphology at two different days of development (E9.5 and E10.5) and measured OFT length and the angle between the proximal and distal regions of the OFT (Fig. 6B). OFT length and angle in *Hes1* ^−/−^ embryos was indistinguishable from that of control littermates at E9.5. In contrast, at E10.5, we observed a significantly increased angle between the proximal and distal regions of the OFT compared to somite-matched control littermates; moreover, OFT length was concomitantly decreased (Fig. 6B). These results demonstrate that the *Hes1* ^−/−^ OFT is shorter and straighter than that of control littermates at midgestation.

### Loss of *Hes1* impairs SHF proliferation without altering differentiation

**Figure 7:**
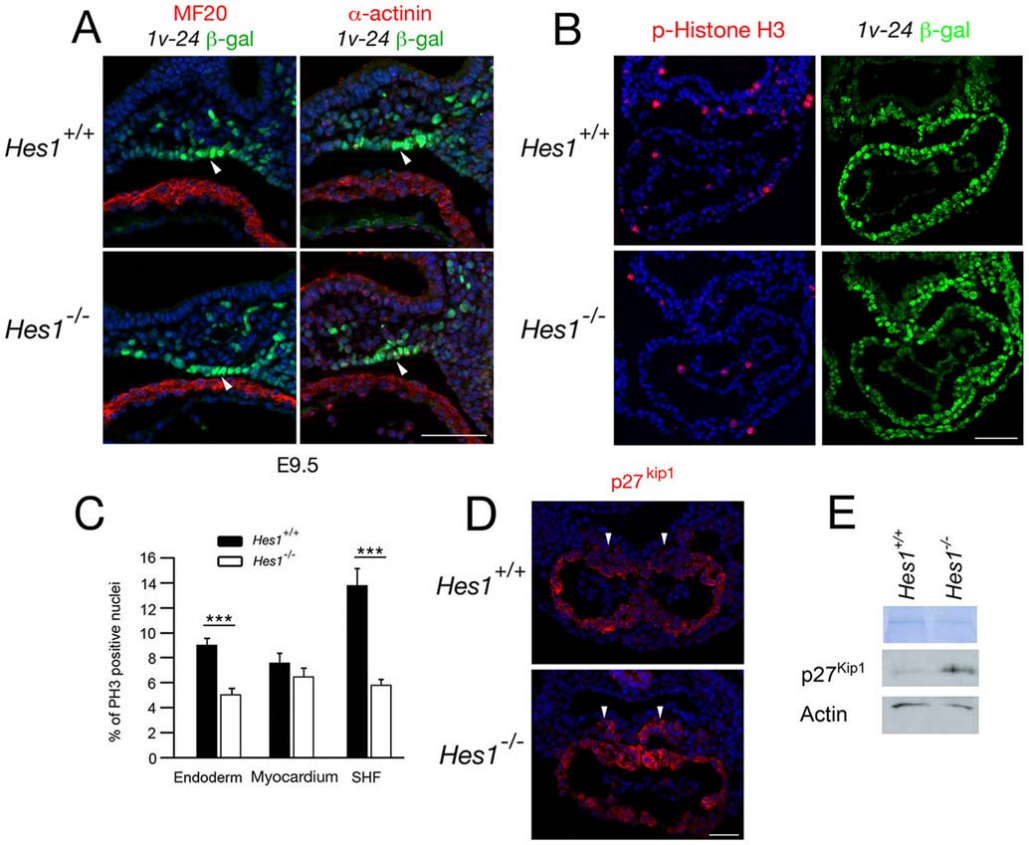
Impact of loss of *Hes1* on differentiation and proliferation in the second heart field. (A) Immunochemistry with anti-MHC (left), anti-α-actinin (right) and anti-β-galactosidase antibodies in transverse sections through the caudal pharyngeal region of an E9.5 embryo carrying the *Mlc1v-nlacZ-24* transgene. Note the β-galactosidase positive α-actinin and MF20 negative cells in the dorsal pericardial wall (arrowheads). Nuclei are labeled with Hoechst (blue). (B) Immunochemistry with anti-phospho-Histone H3 and anti-β-galactosidase antibodies in paraffin sections of E8.5 *Hes1* ^+/+^ and *Hes1* ^−/−^ embryos carrying the *Mlc1v-nlacZ-24* transgene. (C) Histogram comparing the percentage of phospho-Histone H3 positive nuclei in pharyngeal endoderm, myocardium and SHF of *Hes1* ^+/+^ and *Hes1* ^−/−^ embryos. A decrease in phospho-Histone H3 positive nuclei is observed in *Hes1* ^−/−^ SHF and endoderm (p<0.001, Student's t-test). (D) Immunochemistry with an anti-p27^kip1^ antibody in paraffin sections at E8.5. Note that p27^kip1^ is expanded in the SHF of *Hes1* ^−/−^ hearts (arrowheads). Nuclei are labeled with Hoechst (blue). (E) Western blot of microdissected heart and ventral pharyngeal regions of *Hes1* ^+/+^ and *Hes1* ^−/−^ embryos showing elevated p27^kip1^ protein levels in the absence of Hes1. Scale bars (A): 50 µm; (B): 100 µm; (D) 50 µm.

The decrease in OFT length in *Hes1* ^−/−^ embryos is suggestive of a defect in SHF development. Hes1 is known to maintain progenitor cell populations in the developing embryo through the regulation of differentiation and proliferation [22]–[26]. We therefore analyzed SHF differentiation at E9.5; no precocious accumulation of sarcomeric myosin heavy chain or α-actinin was observed in splanchnic mesodermal SHF cells in the dorsal pericardial wall of *Hes1* ^−/−^ embryos at this stage (Fig. 7A). The SHF contributes to both myocardium and smooth muscle at the arterial pole of the heart [2]. Normal myocardial and smooth muscle differentiation at the base of the great arteries were observed in mutant hearts at E12.5 using SM22α and α-actinin immunochemistry (data not shown).

Proliferation within the pharyngeal region of *Hes1* ^−/−^ embryos was evaluated by scoring for the proliferation markers phospho-Histone H3 and Ki67, revealing M-phase and all proliferating cells respectively. SHF progenitors were identified in the dorsal pericardial wall and pharyngeal mesenchyme at E8.5 using Isl1 or β-galactosidase immunochemistry in *Mlc1v-nlacZ-24* embryos. A reduction in the number of phospho-Histone H3 positive (mitotic) *Mlc1v-nlacZ-24* positive cells was observed, from 13.7% in wildtype embryos to 5.7% in *Hes1* ^−/−^ embryos (p<0.001; Fig. 7B, 7C). Moreover, 11.7% of *Mlc1v-nlacZ-24* positive cells were Ki67-negative (non-proliferating) in mutant embryos compared to 5.7% in wildtype embryos (p<0.001; data not shown). A reduction in proliferating cell numbers was also observed in pharyngeal endoderm from 9% in wildtype embryos to 5% in mutant embryos (p<0.001). These results suggest that cell cycle progression in the SHF and pharyngeal endoderm is impaired in mutant embryos. In contrast, no differences in the percentage of phospho-Histone H3 or Ki67 positive cells were observed in the linear heart tube of *Hes1* ^−/−^ embryos at the same E8.5 timepoint. Equivalent low numbers of Caspase 3-positive cells were found in the pharyngeal region of wildtype and mutant embryos, suggesting that apoptosis levels are unaltered in the absence of Hes1 (data not shown).

Hes1 has been shown to control proliferation through the transcriptional repression of cyclin-dependent kinase inhibitors including p27^kip1^ and p57^kip2^ [28], [29]. We investigated p27^kip1^ distribution in the SHF of *Hes1* ^−/−^ embryos and observed an expansion of p27^kip1^ positive cells in SHF cells in the dorsal pericardial wall proximal to the dorsal mesocardium in 3/4 *Hes1* ^−/−^ hearts (Fig. 7D). Immunoblot analysis revealed a 2.15 fold increase in p27^kip1^ levels in microdissected ventral pharyngeal regions including the SHF in the absence of Hes1 (Fig. 7E). Together, these results suggest that loss of Hes1 perturbs proliferation, but not differentiation, of SHF progenitor cells, associated with elevated p27^kip1^levels.

### Cardiac neural crest cell defects in *Hes1* mutant embryos

**Figure 8:**
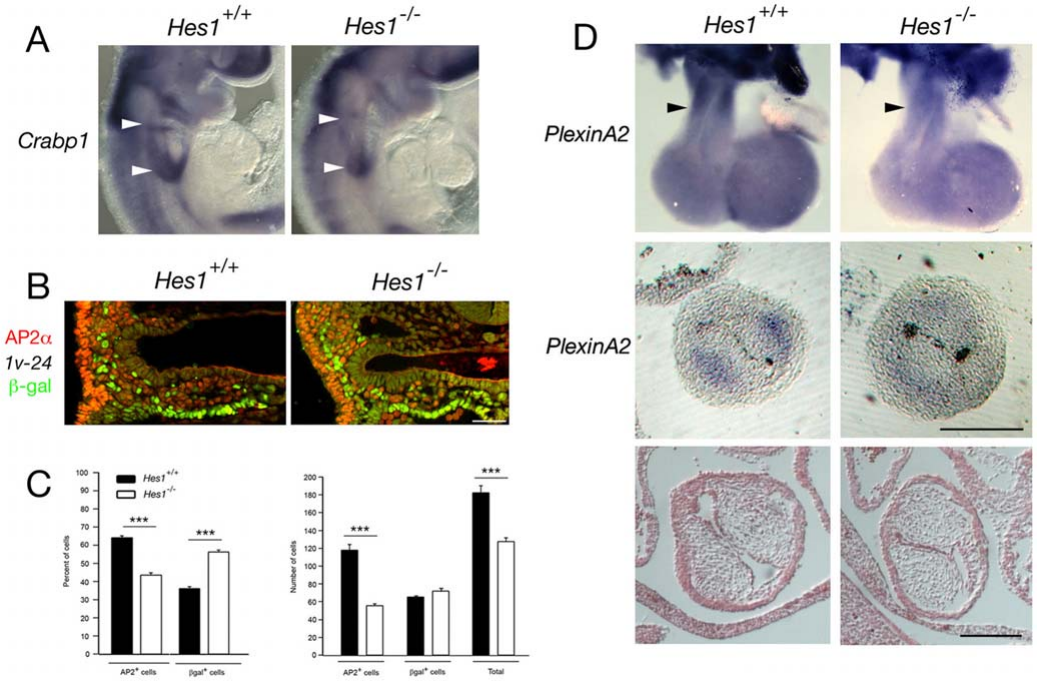
Loss of *Hes1* impairs neural crest development. (A) In situ hybridization at E9.5 showing decreased *Crabp1* transcript accumulation in the caudal pharyngeal region of *Hes1* ^−/−^ compared to *Hes1* ^+/+^ embryos (arrowheads). (B) Immunochemistry with anti-AP-2α (red) and anti-β-galactosidase (green) antibodies in a transverse section through the caudal pharynx of an E9.5 embryo carrying the *Mlc1v-nlacZ-24* transgene. (C) Histograms showing the percentage of β-galactosidase positive nuclei and the percentage of AP-2α positive nuclei in the pharyngeal region of *Hes1* ^+/+^ and *Hes1* ^−/−^ embryos (left) and the numbers of total, β-galactosidase positive and AP-2α positive nuclei (right). Note the decrease in the number of AP-2α positive cells in *Hes1* ^−/−^ embryos (p<0.001, Student's t-test). (D) In situ hybridization at E11.5 showing decreased *PlexinA2* transcript accumulation in the OFT of *Hes1* ^−/−^ compared to *Hes1* ^+/+^ hearts (black arrowheads) in wholemount (top) and transverse sections (middle). Histological analysis reveals normal OFT cushion morphology in *Hes1* ^−/−^ embryos at E11.5 (bottom). Scale bars (B): 50 µm; (D): 200 µm.

Cardiac neural crest cells are critically required for OFT septation and normal SHF deployment [6]. In addition to the above defects in SHF development we observed a reduction in *Crabp1* expression in the region of the 3^rd^ and 4^th^–6^th^ pharyngeal arches in *Hes1* ^−/−^ embryos at E9.5 (Fig. 8A). Transverse sections of the caudal pharynx at E9.5 revealed a reduction in AP-2α positive neural crest cells underlying the pharynx (Fig. 8B). Quantification of neural crest (AP-2α positive) and SHF (*Mlc1v-nlacZ-24* β-galactosidase positive) nuclei revealed a significant decrease in neural crest cell numbers in mutant embryos compared to wildtype littermates (Fig. 8B, 8C). Subsequently, reduced *PlexinA2* transcript accumulation was observed in the distal OFT of *Hes1* ^−/−^ embryos at E11.5 (Fig. 8D). Analysis of OFT cushion morphology at E11.5 revealed normal cushion development in the distal region of the OFT although slight hypocellularity was noted in proximal OFT cushions (Figs 8D, data not shown). Loss of *Hes1* therefore affects two critical cell populations entering the arterial pole of the heart, the SHF and cardiac neural crest cells, leading to impaired heart tube elongation and arterial pole alignment defects at later stages of development.

## Discussion

Here we identify *Hes1* as a transcription factor encoding gene expressed in the SHF and show that Hes1 is required for normal OFT development. Characterization of the insertion site of the *T55* transgene reveals that *Hes1* is the likely endogenous target of regulatory sequences trapped by the transgene. The integration site lies within a region of conserved synteny between mouse and zebrafish that may correspond to a genomic regulatory block, such as have been proposed to contain conserved non-coding elements targeting developmental regulatory genes [30]. Our data suggest that regulatory elements within this block control the spatiotemporal expression pattern of *Hes1*. Comparison of *T55* and *Hes1* expression profiles suggest that the transgene has trapped only a subset of the cis-regulatory elements mediating *Hes1* activation. In contrast, sites of β-galactosidase accumulation but not *Hes1* expression may result from inherent differences in transcript versus β-galactosidase protein stability. Further differences may arise from the fact that *Hes1* is highly regulated both transcriptionally and post-transcriptionally; oscillations in Hes1 protein levels play a critical role in measuring developmental time in unsegmented paraxial mesoderm [21]. β-galactosidase activity in a subregion of OFT myocardium may thus in part provide a readout of prior heterogeneity in *Hes1* expression in SHF progenitor cells.

Hes1 is known to be a downstream component of the Notch signaling pathway and a critical regulator of multiple stem cell populations in the developing embryo [22]. Accelerated differentiation in *Hes1* mutant embryos results in depletion of progenitor cell populations and a failure of late differentiating cell types [22]–[26]. Here we show that Hes1 is required for normal deployment of SHF progenitor cells. However, we did not observe accelerated differentiation in the SHF of *Hes1* ^−/−^ embryos. A possible explanation lies in the observation that *Hey1* and *Hey2* transcripts are also detectable in pharyngeal mesoderm (data not shown). Hes1 can heterodimerize with Hey1 to effect strong transcriptional repression and *Hes1*, *Hes3* and *Hes5* are known to have overlapping functions in the developing nervous system, uncovered by analysis of compound null embryos [31], [32]. Hes1 may therefore overlap in function with other members of the Hes and Hey gene families to regulate SHF development. In support of this argument, overriding aorta has been observed in embryos lacking Hey2 [17]–[20].

Hes1 also maintains progenitor cell populations in the developing embryo by regulating proliferation. In particular, Hes1 represses transcription of the CDK inhibitors p27^kip1^ and p57^kip2^ [28], [29]. We observed that levels of the mitotic marker phospho-Histone H3 are reduced in the SHF of *Hes1* mutant embryos at E8.5, whereas the number of Ki67-negative quiescent cells is increased. p27^kip1^ protein distribution at this stage reflects the low proliferative nature of differentiated cells in the linear heart tube. We observed that p27^kip1^ was expanded and upregulated in the dorsal pericardial wall of *Hes1* mutant embryos, consistent with release from Hes1 mediated repression and providing new mechanistic insights into the regulation of SHF proliferation.

In addition to reduced proliferation in the SHF, the *Hes1* cardiac phenotype is likely to be compounded by the observation that less neural crest cells are found in the ventral pharyngeal region and distal OFT of mutant embryos. *Hes1* ^−/−^ embryos have defects in neural development that may affect the number of migrating neural crest cells [23] and low-level *Hes1* transcript accumulation is observed in neural crest cells in the distal OFT. Alternatively, loss of endodermal or SHF signals may indirectly impact on neural crest cell numbers. Neural crest ablation in avian embryos results in impaired SHF deployment and a shortened OFT [6], [8]; reduction of neural crest in the caudal pharynx could thus in turn influence SHF development in *Hes1* ^−/−^ embryos. OFT alignment defects in *Hes1* mutant embryos have been independently observed by another group who, in addition, noted aortic arch artery abnormalities associated with a defective smooth muscle contribution at E11.5 (P. Scambler, personal communication). Neural crest cell anomalies may therefore also contribute directly to the arterial pole phenotype of *Hes1* mutant embryos. While we did not observe defects in great artery smooth muscle development, slight hypocellularity of OFT cushions could result from reduced neural crest cell numbers. Alternatively, epithelial-mesenchymal transition of OFT endothelial cells, known to be regulated by Notch signalling [9], may be defective in *Hes1* ^−/−^ embryos, possibly due to a later role of *Hes1* in the OFT itself. Dissecting the requirement for *Hes1* in different cell-types by conditional mutagenesis will determine the relative importance of different sites of *Hes1* expression (including pharyngeal mesoderm, endoderm, ectoderm, neural crest and endothelial cells) during OFT morphogenesis.

The early defect in OFT elongation is likely to underlie the subsequent ventriculoarterial alignment defects observed in fetal *Hes1* ^−/−^ hearts. Intriguingly, a dextraposed aorta and ventricular septal defect are observed in less than half of mutant embryos whereas a shorter OFT was observed in all mutant embryos scored at E10.5. Given that there is no loss of *Hes1* ^−/−^ embryos between E10.5 and E15.5, this suggests that there is a degree of phenotypic recovery. The embryonic OFT obtains its maximal length at E10.5 and subsequently becomes incorporated into the ventricular outlets and non-myocardial base of the great arteries; OFT length in *Hes1* ^−/−^ embryos may be on the borderline of that required to achieve normal alignment.

Overriding aorta and ventricular septal defects are common components of human congenital heart defects. Defects in OFT development in human patients carrying *JAGGED1* or *NOTCH2* mutations or in mice carrying mutations in Notch ligands, receptors or target genes demonstrate the importance of this intercellular signaling pathway in arterial pole development [13]–[20]. Notch signaling has also been implicated in neural crest and valve development [9], [12], [16]. The regulation of SHF progenitor cell deployment during heart tube extension may be an additional mechanism by which Notch intercellular signaling controls heart development. Future investigation of the upstream control of *Hes1* expression in the SHF will test this hypothesis.

## Materials and Methods

### Mice

*A17-Myf5-nlacZ-T55* (*T55*) and *Mlc1v-nlacZ-24* transgenic mice have been previously described [3], [27]. *Hes1^+/−^* mice were kindly provided by François Guillemot (NIMR, UK) and maintained on a CD1 outbred background [23]. DNA isolated from tail-tips or yolk sacs was genotyped for *Hes1* alleles as described [33]. *Mlc1v-nlacZ-24* and *A17-Myf5-nlacZ-T55* transgenic mice were genotyped by X-gal staining or by PCR [3], [27]. Mouse care and procedures were in accordance with guidelines approved by the Departmental Direction of Veterinary Services of the French Ministry of Agriculture.

### Isolation of a transgene integration site junction fragment

Inverse PCR was performed using *SphI* and *Bgl*II digested *T55* hemizygous tail-tip DNA, as described [3]. Primers A and B were used for inverse PCR using the Expand PCR system (Roche). A 2.6 kb PCR product containing 256 bp of flanking sequence was subcloned using the pGemT-Easy vector (Promega). DNA sequences were aligned using NCBI BLAST, DNA Strider (Release 1.4) and the Ensembl mouse genome browser. Southern blot mapping was carried out with a 770 bp probe generated by PCR using primers C and D that detects a 3.8 kb wildtype fragment and a 5.5 kb fragment subsequent to transgene integration in *Bam*HI digested genomic DNA. PCR validation was carried out using primers E, F and G. Primers E and F generate a 500 bp product from the wildtype locus and F and G a 374 bp product after transgene integration.

Oligonucleotide primer sequences

A 5′-AAAACGACGGGATCATCGCGAGCCATGC-3′
B 5′-ACTGGAGGCTGAAGTTCAGATGTGCGGC-3′
C 5′-GGTGGCAGCAGTAATCCATGATCTTG-3′
D 5′-TGTCAAATCAGATCTCTCCAGATGCC-3′
E 5′-CCTGCTGCTTTGGACAATGC-3′
F 5′-AAGTCGTTGGAGACCTTGTC-3′
G 5′-ACCGCTGGATAACGACATTG-3′

### Wholemount analysis, histology and immunochemistry

Embryos were dated taking the day of the plug as embryonic day (E) 0.5. Hearts were dissected at E15.5 and analyzed using a Zeiss Lumar stereo dissecting microscope; selected hearts were subsequently analyzed after cryostat or paraffin embedding and sectioning using standard techniques. Sections were stained with haemotoxilin and eosin or treated for 15 min with antigen unmasking solution (Vector) prior to immunohistochemistry using standard protocols. Primary antibodies used were: β-galactosidase (1/300, Cappel), MF20 (1/20, DSHB), AP-2α (1/25, DSHB clone 3B5), α-actinin (1/200, DSHB), p27^kip1^ (1/200, Cell Signaling), phospho-Histone H3 (1/400, Upstate), Ki67 (1/25, Dako), Isl1 (1/100, DSHB clones 402D6 and 394D5) and Caspase 3 (1/100, Cell Signaling). Secondary antibodies used were: anti-mouse Alexa 488 (Interchim), anti-mouse Cy3 (Jackson), anti-rat biotin (Jackson), anti-rabbit Alexa 488 (Interchim) and anti-rabbit Cy3 (Jackson). All secondary antibodies were used at a 1/200 dilution. The anti-rat biotinylated secondary was amplified using the Renaissance TSA Fluorescence System (Perkin Elmer), according to the manufacturer's instructions. Slides were counterstained with Hoechst and observed using an ApoTome microscope (Zeiss). Mesenchymal Ap-2α positive nuclei were scored at E9.5 in 5 or more 7 µm sections, 14 µm apart, from 3 mutant and 3 control embryos. X-gal staining was carried out as described [3]; embryos were collected and fixed for 10 min in 4% paraformaldehyde, extensively washed in 1× PBS and stained for 5 hours at 37°C in a solution containing 4 mg/ml of X-gal. After staining, the samples were washed in PBS, post-fixed and observed under a Zeiss Lumar stereomicroscope. For each experiment a minimum of 3 embryos of each genotype was scored. OFT length and angle were measured from stereotyped ventral views of X-gal stained *Mlc1v-nlacZ-24* embryos at E9.5 (24–26 somites) and E10.5 (30–35 somites) using Metamorph software.

### Immunoblotting

Protein lysates were obtained from microdissected E8.5 hearts and ventral pharyngeal regions of control and mutant embryos. Proteins were separated by SDS-PAGE and transferred to Hybond-C extra membranes (Amersham Biosciences) prior to blocking with 5% dry milk for 2 hours. Membranes were incubated overnight at 4°C with primary antibodies (anti-actin, diluted 1/2000, Sigma; anti-p27kip1, diluted 1/1000, Cell Signaling Technology) followed by chemiluminescent detection (Western Lightening, Enhanced Luminol reagent, Perkin Elmer). Quantification of immunoblots was carried out using Image Quant TL software (Amersham Biosciences).

### In situ hybridization

Whole mount in-situ hybridization was performed as previously described [3]. The *Hes1* riboprobe was synthesised by T7 RNA polymerase from a 553 bp template amplified by PCR using primers Hes1F and Hes1R:

Hes1F 5′-GAGAGGCTGCCAAGGTTTTT-3′
Hes1R 5′-GTAATACGACTCACTATAGGGTCAAATAAACTTCCCCAAAGGA-3′

The following riboprobes were prepared from plasmid templates: *Crabp1* [34], Fgf10 [3], *Isl1* [35], *PlexinA2* [36], *lacZ* [3]. Riboprobes for *Opa1*, *953002-Rik*, *Gm1968*, *Hey1* and *Hey2* were generated using T7 RNA polymerase from PCR amplified templates. Primer details are avaliable on request. For each experiment a minimum of two embryos per genotype were scored.

### Fluorescent in situ hybridization

Fluorescent in situ hybridization was carried out as described [37]. Briefly, metaphase spreads were prepared from male mice carrying the *T55* transgene. Concanavalin A-stimulated splenic cells were cultured for 72 hours with 5-BrdU added for the final 6 hours of culture (60 mg/ml of medium) to ensure chromosomal R-banding. The biotinylated *lacZ* probe was mixed with hybridization solution at a final concentration of 50 mg/ml and used at 200 ng per slide after competition of aspecific repetitive sequences. The hybridized probe was detected by fluorescent isothiocyanate-conjugated avidin (Vector laboratories). Chromosomes were counterstained with propidium iodide. A total of 50 metaphase cells were analyzed.

### Cell proliferation and apoptosis analysis

Proliferation was evaluated by immunochemical detection of phospho-Histone H3 and Ki67 at E8.5. Data were obtained from at least four sections per embryo for three embryos of each genotype. Equivalent total cell numbers were scored in wildtype and homozygous null embryos. For phospho-Histone H3 mean total cell numbers counted per section were 56.1 (endoderm), 59.6 (heart) and 49.4 (*Mlc1v-nlacZ-24* transgene positive SHF cells) for wildtype embryos and 59.2 (endoderm), 52.3 (heart) and 42.5 (*Mlc1v-nlacZ-24* transgene positive SHF cells) for *Hes1* ^−/−^ embryos. For Ki67 mean total *Mlc1v-nlacZ-24* transgene positive SHF cell numbers counted per section were 62 for wildtype embryos and 64 for *Hes1* ^−/−^ embryos. Statistical analysis was carried out using Student's t-test. Cell death was evaluated at E9.5 using a Caspase 3 antibody and scored as above.
